# D‐Ser2‐oxyntomodulin ameliorated Aβ31‐35‐induced circadian rhythm disorder in mice

**DOI:** 10.1111/cns.13211

**Published:** 2019-08-14

**Authors:** Li Wang, Jin Zhao, Chang‐Tu Wang, Xiao‐Hong Hou, Na Ning, Cong Sun, Shuai Guo, Yuan Yuan, Lin Li, Christian Hölscher, Xiao‐Hui Wang

**Affiliations:** ^1^ Department of Pathology Shanxi Medical University Taiyuan China; ^2^ Laboratory of Chronobiology Shanxi Medical University Taiyuan China; ^3^ Laboratory of Morphology, Department of Basic Medical Sciences Shanxi Medical University Taiyuan China; ^4^ Key Laboratory of Cellular Physiology Shanxi Medical University Taiyuan China; ^5^ Second Hospital Shanxi Medical University Taiyuan China; ^6^ Biomedical and Life Science, Faculty of Health and Medicine Lancaster University Lancaster UK

**Keywords:** Alzheimer's disease, Bmal1, glucagon‐like peptide‐1, Per2

## Abstract

**Introduction:**

The occurrence of circadian rhythm disorder in patients with Alzheimer's disease (AD) is closely related to the abnormal deposition of amyloid‐β (Aβ), and d‐Ser2‐oxyntomodulin (Oxy) is a protease‐resistant oxyntomodulin analogue that has been shown to exert neuroprotective effects.

**Aims:**

This study aimed to explore whether Oxy, a new GLP‐1R/GCGR dual receptor agonist, can improve the Aβ‐induced disrupted circadian rhythm and the role of GLP‐1R.

**Methods:**

A mouse wheel‐running experiment was performed to explore the circadian rhythm, and western blotting and real‐time PCR were performed to assess the expression of the circadian clock genes *Bmal1* and *Per2*. Furthermore, a lentivirus encoding an shGLP‐1R‐GFP‐PURO was used to interfere with GLP‐1R gene expression and so explore the role of GLP‐1R.

**Results:**

The present study has confirmed that Oxy could restore Aβ31‐35‐induced circadian rhythm disorders and improve the abnormal expression of Bmal1 and Per2. After interfering the GLP‐1R gene, we found that Oxy could not improve the Aβ31‐35‐induced circadian rhythm disorder and abnormal expression of clock genes.

**Conclusion:**

This study demonstrated that Oxy could improve Aβ31‐35‐induced circadian rhythm disorders, and GLP‐1R plays a critical role. This study thus describes a novel target that may be potentially used in the treatment of AD.

## 1 INTRODUCTION

The circadian rhythm refers to a period of approximately 24 hours that occurs spontaneously in vivo. Circadian rhythm disorders often occur in the course of various neurodegenerative diseases, such as Alzheimer's disease (AD).^1^ AD patients exhibit abnormal sleep patterns, such as daytime sleepiness or sleep fragmentation,^2^ and the occurrence of circadian rhythm disorders in AD patients is closely related to the abnormal deposition of amyloid β‐protein (Aβ).^3^ Aβ deposition, circadian rhythm disorder, and abnormal expression of the Bmal1 and Per2 genes have been observed in the brains of 5XFAD mice.^4^ Previously, we reported that direct intrahippocampal injections of Aβ lead to significant circadian rhythm abnormalities in mice.^5^ However, there is currently no effective treatment for Aβ‐induced circadian rhythm disorders.

Recent evidence has indicated a significant correlation between type 2 diabetes (T2DM) and AD,^6^ and glucagon‐like peptide‐1 (GLP‐1), a therapeutic drug of T2DM, has been shown to impart significant neuroprotection. We found that the GLP‐1 receptor (GLP‐1R) agonist Exendin‐4 improves Aβ31‐35‐induced circadian rhythm disorders.^5^ However, the study showed that during hyperglycemia, the activation of the GLP‐1R could induce acute blood glucose‐lowering effects.^7^ Therefore, it is essential to identify safer and more effective treatments. Oxyntomodulin (OXM), a natural gastrointestinal hormone, activates both the GLP‐1R and glucagon receptor (GCGR), thus imparting a stronger effect than a single‐receptor agonist in promoting insulin secretion.^8^ The activation of GCGR by OXM could effectively prevent acute hypoglycemia that is mediated by GLP‐1R activation.^7^ D‐Ser2‐Oxyntomodulin (Oxy) is a protease‐resistant oxyntomodulin analogue that has a longer half‐life of 12 hours 9 and remains a balanced agonist at both receptors.^10^ Oxy can reverse MPTP‐induced dyskinesia in a Parkinson's disease mouse model and impart neuroprotection.^11^ More importantly, OXM was found to be involved in food‐induced reset of the liver clock by regulating the expression of the clock genes Per1 and Per2.^12^ However, whether it can improve the Aβ‐induced circadian rhythm disorders remains unknown.

The expression level of GLP‐1R in the mouse brain is higher than that of GCGR.^13^ Accordingly, we speculate that the role of Oxy in the brain largely depends on its activation by GLP‐1R. Studies have shown that Oxy promotes neuronal growth by antagonizing the Aβ1‐42‐induced reduction in rat hippocampal neuronal cell survival, and the phenomenon could be attenuated by the GLP‐1R antagonist Exendin (9‐39).^14^ Therefore, we hypothesize that Oxy improves Aβ‐induced circadian rhythm disorders mainly by activating GLP‐1R and its downstream pathways.

In this study, we investigated whether Oxy ameliorates Aβ31‐35‐induced circadian rhythm disorders and identified the role of GLP‐1R using lentiviral vectors that interfere GLP‐1R expression both in vivo and in vitro to provide a novel therapeutic target for the treatment of AD.

## 2 METHODS

### 2.1 Experimental animals and reagents

Six‐to‐eight‐week‐old male C57BL/6 mice (18‐22 g) were obtained from the Research Animal Center of Shanxi Medical University with approval of the Shanxi Animal Research Ethics Committee, and the procedures were performed in accordance with ethical guidelines. The approval code is SYXK2015‐0001. The mice were placed in an environment with appropriate temperature and humidity, and food was provided ad libitum during the entire study.

The main reagents for the experiment included Aβ31‐35 (Abcam), D‐Ser2‐oxyntomodulin (provided by Professor Christian Hölscher), Bmal1 antibody (Abcam), Per2 antibody (Santa Cruz Biotechnology), and GLP‐1R antibody (Abcam).

### 2.2 Intrahippocampal injection

C57BL/6 mice were anesthetized intraperitoneally with 5% chloral hydrate and fixed on a standard stereotaxic instrument. According to the mouse anatomical atlas, the bregma was exposed and used as a benchmark to search for the bilateral hippocampus of mice (2.0 mm posterior to the bregma, 1.8 mm lateral to the midline, and 1.8 mm below the skull surface). The mice were randomly divided into four groups: the control group, which received the equal doses of vehicle sterile water via hippocampal injection; Aβ31‐35 group, which received 750 nmol/kg of Aβ31‐35 via hippocampal injection; Oxy pretreatment group (Oxy + Aβ31‐35 group), which were pretreated with Oxy (30 nmol/kg) via hippocampal injection and 15 minutes later received Aβ31‐35; and Oxy alone treatment group (Oxy group), which received 30 nmol/kg of Oxy via hippocampal injection.

### 2.3 Wheel‐running behavior test

Each group of mice was placed on a wheel‐running device. The light setting of the cages was 12 hours light:12 hours dark (LD) for 1 week to develop a regular sleep‐exercise pattern, then converted to constant darkness (DD) for two weeks to examine the effects of Aβ31‐35 or/and Oxy on the endogenous rhythm. The time during the DD conditions was referred to as the circadian time (CT), and the onset of activity was defined as circadian time 12 (CT12). Upon termination of wheel running, the mice were decapitated at CT4, CT8, CT12, CT16, CT20, and CT24, and then, the hippocampus was immediately isolated and placed on ice in a dark environment. Running wheel activity was recorded using the VitalView program and analyzed using ActiView software.

### 2.4 Cell culture and administration

The mouse hippocampal HT22 cells (Ginio Biotechnology Co., Ltd.) were cultured with DMEM complete medium containing 10% fetal bovine serum and 1% penicillin‐streptomycin antibiotics in an incubator at 37°C with 5% CO_2_ and maximal humidity. Cell synchronization was performed when the cells had completely attached as follows: the culture medium was replaced with a starvation medium containing 1% fetal bovine serum and 1% penicillin‐streptomycin antibiotics. After 1 hour starvation, the circadian rhythm of the HT22 cells was regarded as synchronized with circadian time 0 (CT0). The synchronized cells were cultured in complete medium again for n hours as CT n. The control group was cultured in the complete medium; the Aβ group was cultured in the complete medium containing Aβ31‐35 (5 μmol/L); the Oxy + Aβ group was pretreated with Oxy for 1 hour, and then, Aβ31‐35 was added at the same doses as earlier described; and the Oxy group was cultured in the complete medium with Oxy (100 nmol/L). The cells were collected at CT4, CT8, CT12, CT16, CT20, and CT24.

### 2.5 Real‐time PCR analysis

Total RNA was extracted using the TRIzol method and then reversed transcribed into cDNA. The SYBR Green kit was used, and the corresponding primers were employed for specific amplification. Primers were designed as follows: Bmal1 (GenBank Accession number NM_001243048.1), forward primer: 5′‐ACGACATAGGACACCTCGCAGA‐3′, reverse primer: 5′‐TCCTTGGTCCACGGGTTCA‐3′; Per2 (GenBank Acc. No. NM_011066.3), forward primer: 5′‐TGGTCTGGACTGCACATCTGG3′, reverse primer: 5′‐AGGTCACTTGACGT‐GGAGATGG‐3′; and GAPDH (GenBank Acc. No. NM_008084.2), forward primer: 5′‐AAATGGTGAAGGTCGGTGTGAAC‐3′, reverse primer: 5′‐CAACAATCTCCACTTTGCCACTG‐3′. All results were normalized to that of GAPDH at CT4. The relative gene expression was determined using the 2^−ΔΔCT^ comparative method.

### 2.6 Western blot analysis

RIPA lysis buffer was added to the collected samples for protein extraction, and protein concentrations were measured using the BCA protein assay kit. After boiling the samples to denature the proteins, SDS‐PAGE was performed, and then the resulting protein bands were transferred onto a PVDF membrane. The membrane was blocked for 2 hours at room temperature in TBST containing 5% nonfat milk powder. The membrane was then incubated with the primary antibody overnight at 4°C and subsequently with the corresponding secondary antibody for 2 hours after TBST washing. The membrane was washed, and the image was captured using a gel imaging system. ImageJ was used to analyze and calculate the relative expression of the protein.

### 2.7 GLP‐1R interference and efficiency detection in mice hippocampus

The shGLP‐1R plasmid was constructed by Hanbio Biotechnology Co., Ltd., containing lentivirus with GFP and puromycin‐resistant gene (LV‐shGLP‐1R‐GFP‐PURO). Hippocampal tissues were sliced into 30‐μm sections after intrahippocampal injection of LV‐shGLP‐1R‐GFP‐PURO, the slides were observed under a laser scanning confocal microscope, and interference efficiency of GLP‐1R was determined using western blot and immunohistochemical staining.

### 2.8 Immunohistochemical staining

Seven days after injection of LV‐GLP‐1R lentivirus and LV‐NC (nonspecific shRNA lentivirus) into the hippocampus of mice, brain tissues were isolated and fixed with 10% neutral formalin solution, followed by paraffin embedding and tissue sectioning. The sectioned tissue slices were dried in an oven, deparaffinized in xylene solution, and conventionally hydrated, followed by antigen retrieval, blocking for 10 minutes with 10% goat serum at room temperature, and incubation with GLP‐1R antibodies overnight at 4°C. The sections of the hippocampal tissues were then incubated with the corresponding secondary antibodies at 37°C for 20 minutes, and the signals were subsequently developed in 3,3’‐diaminobenzidine (DAB) substrate solution, followed by hematoxylin counterstaining, 1% hydrochloric acid alcohol‐differentiation, dried across an ethanol gradient, cleared with a xylene solution, and mounted in neutral gum.

### 2.9 GLP‐1R gene silencing and detection in HT22 cells

The suitable lentivirus and the auxiliary agent polybrene were used according to the set of a multiplicity of infection (MOI) and added to the cells. Approximately 12‐16 hours after virus infection, the virus‐containing medium was replaced with a fresh complete medium. The virus‐infected cells were screened after 72 hours as follows: uninfected and virus‐infected cells with a uniform cell density were prepared for pre‐screening, and the cells were cultured in a complete medium containing puromycin at the appropriate concentration. The culture medium was replaced every other day until all uninfected cells had died. Then, the virus‐infected cells were further cultured for another 1‐2 days with half the concentration of puromycin. The percentage of GFP‐positive cells was observed under laser scanning confocal microscope to assess the infection efficiency. Then, the GLP‐1R expression was determined using immunofluorescence and Western blotting.

### 2.10 Immunofluorescence

The mouse brains were sectioned at a thickness of 30 μm. The hippocampal tissue sections were incubated in an oven overnight at 37°C and then rinsed thrice with PBS. The HT22 cells were evenly inoculated onto slides and fixed with 4% paraformaldehyde for 20 minutes. Slides containing mouse brain tissues and slides containing HT22 cells were incubated with 10% goat serum for 1 hour and 30 minutes, respectively. Next, the slides were incubated overnight at 4°C with GLP‐1R primary antibodies. The next day, the slides were incubated with fluorescent secondary antibodies at 37°C for 2 hours or 1 hour. After sealing, the images were captured under a laser scanning confocal microscope.

### 2.11 Statistical analysis

Statistical analysis was performed with SPSS Version 16.0 statistical software package. Data were expressed as the mean ± SEM. JTK_CYCLE was used to analyze the rhythmic expression of clock genes.^15^ One‐way ANOVA was used for comparison among multiple groups, and the LSD‐*t* test was used for comparison between groups. The significance level of *α* was set at .05. *P* < .05 was considered statistically significant.

## 3 RESULTS

### 3.1 Oxy ameliorated Aβ31‐35‐induced circadian rhythm disorders in mice

**Figure 1:**
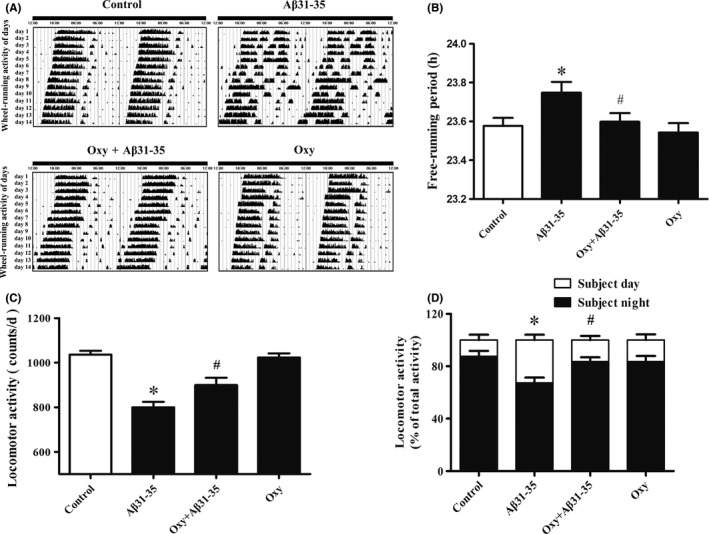
The influence of Oxy on Aβ31‐35‐induced circadian rhythm disorder in mice. A, Representative locomotor activity records of each group. B, The free‐running period of the locomotor activity rhythm in each group. C, The effect of Oxy on Aβ31‐35‐induced decrease in locomotor activity. D, The ratio of the subjective night activity/total activity in each group. Data were expressed as mean ± SEM (n = 10 per group). *P < .05 compared to the control group; ^#^ *P* < .05 compared to the Aβ31‐35 group

The circadian rhythm wheel‐running activity of the control mice mostly occurred at night (Figure 1A). However, the circadian rhythm wheel‐running activity of mice injected with Aβ31‐35 was irregular, which was due to a disruption in the daily sleep‐wake cycle (Figure 1A). The free‐running period was significantly prolonged (Figure 1B), and locomotor activity was significantly reduced (Figure 1C). The ratio of the subjective night activity to the total activity significantly decreased (Figure 1D). Furthermore, pretreatment with Oxy improved the Aβ31‐35‐induced disruption of the circadian rhythm. The sleep‐wake cycle, free‐running period, locomotor activity, and ratio of the subjective night activity/total activity were significantly recovered (Figure 1). There was no significant difference between the Oxy‐treatment group and the control group (Figure 1). These results suggest that Oxy improves Aβ31‐35‐induced circadian rhythm disorder.

### 3.2 Oxy improves the Aβ31‐35‐induced abnormal expression of Bmal1 and Per2 in the mouse hippocampus

**Figure 2:**
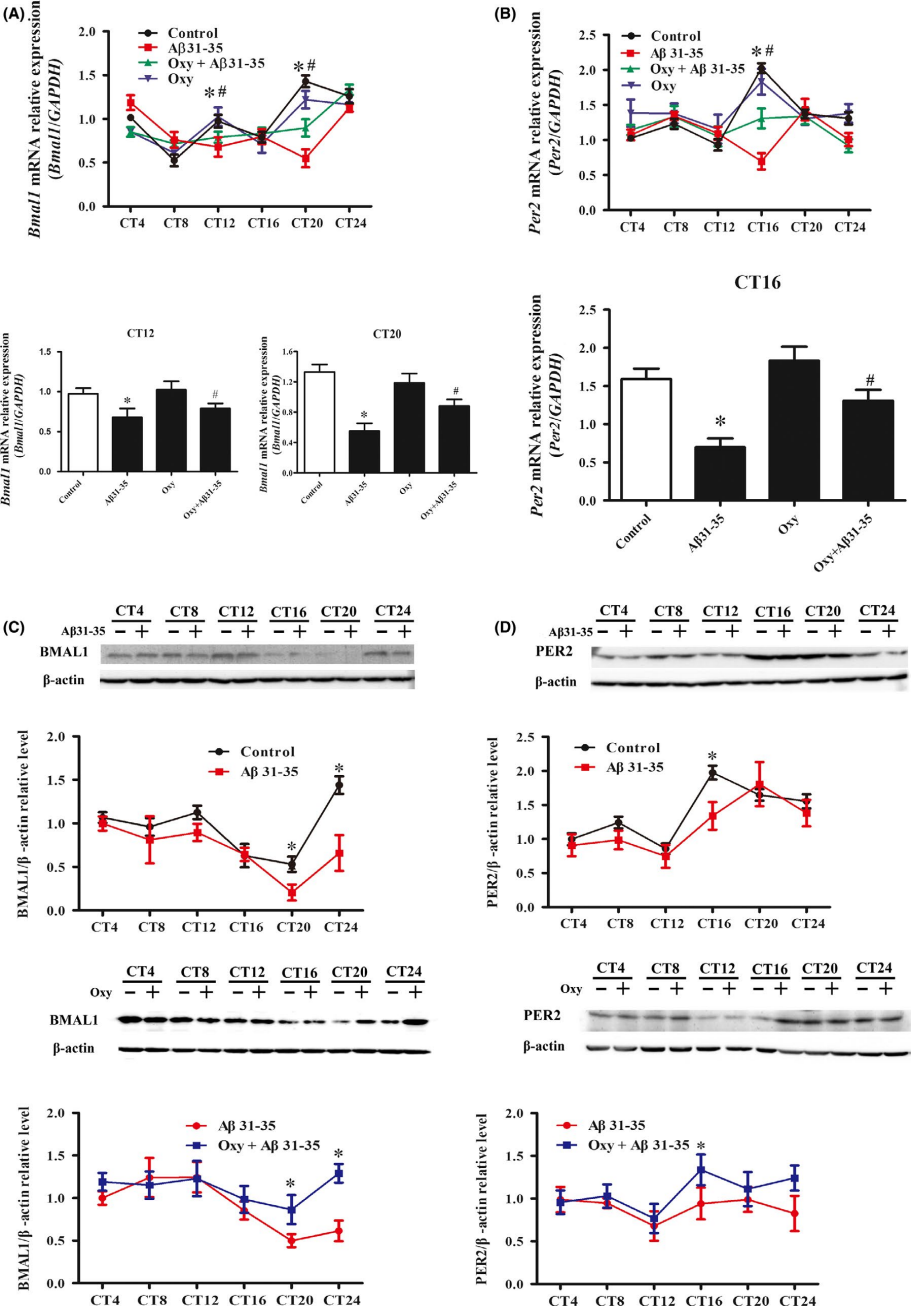
The effect of Oxy on Aβ31‐35‐induced abnormal expression of Bmal1 and per2 in the mouse hippocampus. A, B, mRNA levels of *Bmal1* and *Per2* were assessed at the indicated circadian times. C, D, Representative immunoblots showed the levels of BMAL1 and PER2. Western blotting was performed using β‐actin as loading control. The data were expressed as the mean ± SEM (n = 6 per group). *P < .05 compared to the control group; ^#^ *P* < .05 compared to the Aβ31‐35 group

JTK_CYCLE was used to analyze rhythmic mRNA and protein expression of Bmal1 and Per2. The results showed that the mRNA and protein expression of Bmal1 and Per2 in the hippocampus of the control group exhibited a prominent circadian rhythm. Aβ31‐35 disrupted the circadian oscillation of *Bmal1* mRNA and *Per2* mRNA, as indicated by a decreased expression at CT12, CT20 (*Bmal1* mRNA), and CT16 (*Per2* mRNA) (Figure 2A,B). The mRNA levels of *Bmal1* at CT12/CT20 and *Per2* at CT16 showed a significant increase after pretreatment with Oxy compared with the Aβ31‐35 group. There was no significant difference in the levels of *Bmal1* and *Per2* mRNA expression between the Oxy alone group and control group (Figure 2A,B). BMAL1 protein expression was significantly lower in the Aβ31‐35 group than in the control group at CT20 and CT24 (Figure 2C), and PER2 protein expression significantly decreased in the Aβ31‐35 group at CT16 (Figure 2D). In addition, a partial recovery of the BMAL1 expression at CT20/CT24 and the PER2 expression at CT16 was observed after the Oxy pretreatment (Figure 2C,D).

### 3.3 Oxy improves the Aβ31‐35‐induced abnormal expression of Bmal1 and Per2 in hippocampal HT22 cells

**Figure 3:**
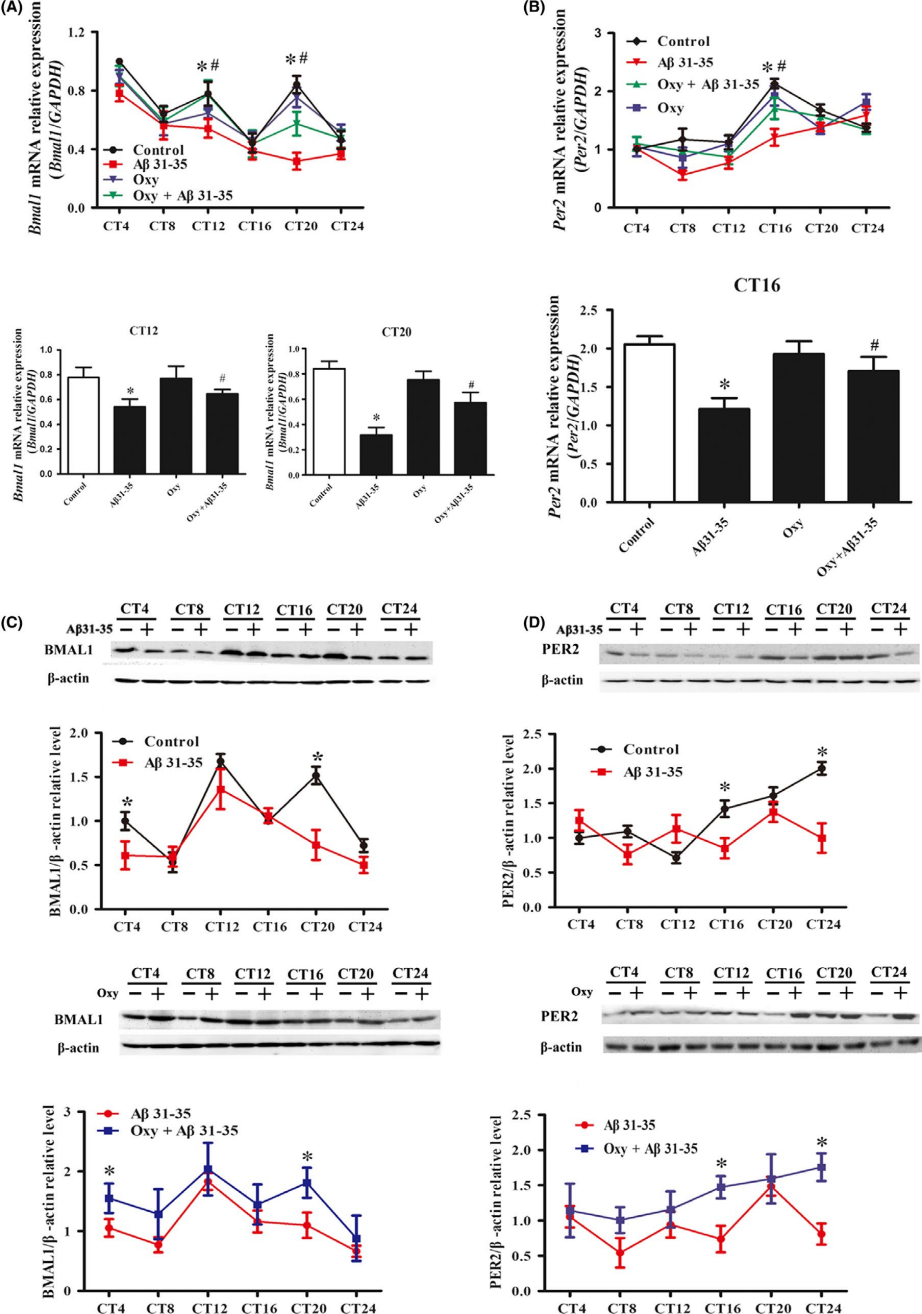
The impact of Oxy on Aβ31‐35‐induced abnormal expression of Bmal1 and Per2 in hippocampal HT22 cells. A, B, Real‐time PCR was used to measure *Bmal1* and *Per2* mRNA expression in HT22 cells. C, D, Western blotting analysis showing the protein expression of BMAL1 and PER2. Data were expressed as the mean ± SEM (n = 6 per group). *P < .05 compared to the control group; ^#^ *P* < .05 compared to the Aβ31‐35 group

Rhythmic mRNA and protein expression of Bmal1 and Per2 in HT22 cells were analyzed using JTK_CYCLE. Bmal1 and Per2 mRNA and protein expression exhibited circadian oscillation in the control group. Aβ31‐35 significantly reduced the *Bmal1* mRNA expression at CT12 and CT20 and decreased the mRNA expression of *Per2* at CT16 (Figure 3A,B). Then, Western blotting was performed to assess the BMAL1 protein expression in each group. A significant decrease in the BMAL1 protein expression at CT4/CT20 and PER2 protein expression at CT16/CT24 was found in Aβ31‐35‐treated HT22 cells (Figure 3C,D). The mRNA and protein expression of Bmal1 and Per2 was partly restored after pretreatment with Oxy (Figure 3). No significant differences between the Oxy‐treatment group and control group were observed at each time point (Figure 3). These results indicate that Oxy restores the Aβ31‐35‐induced abnormal expression of Bmal1 and Per2 in HT22 cells.

### 3.4 Oxy restores Aβ31‐35‐induced downregulation of GLP‐1R in HT22 cells

**Figure 4:**
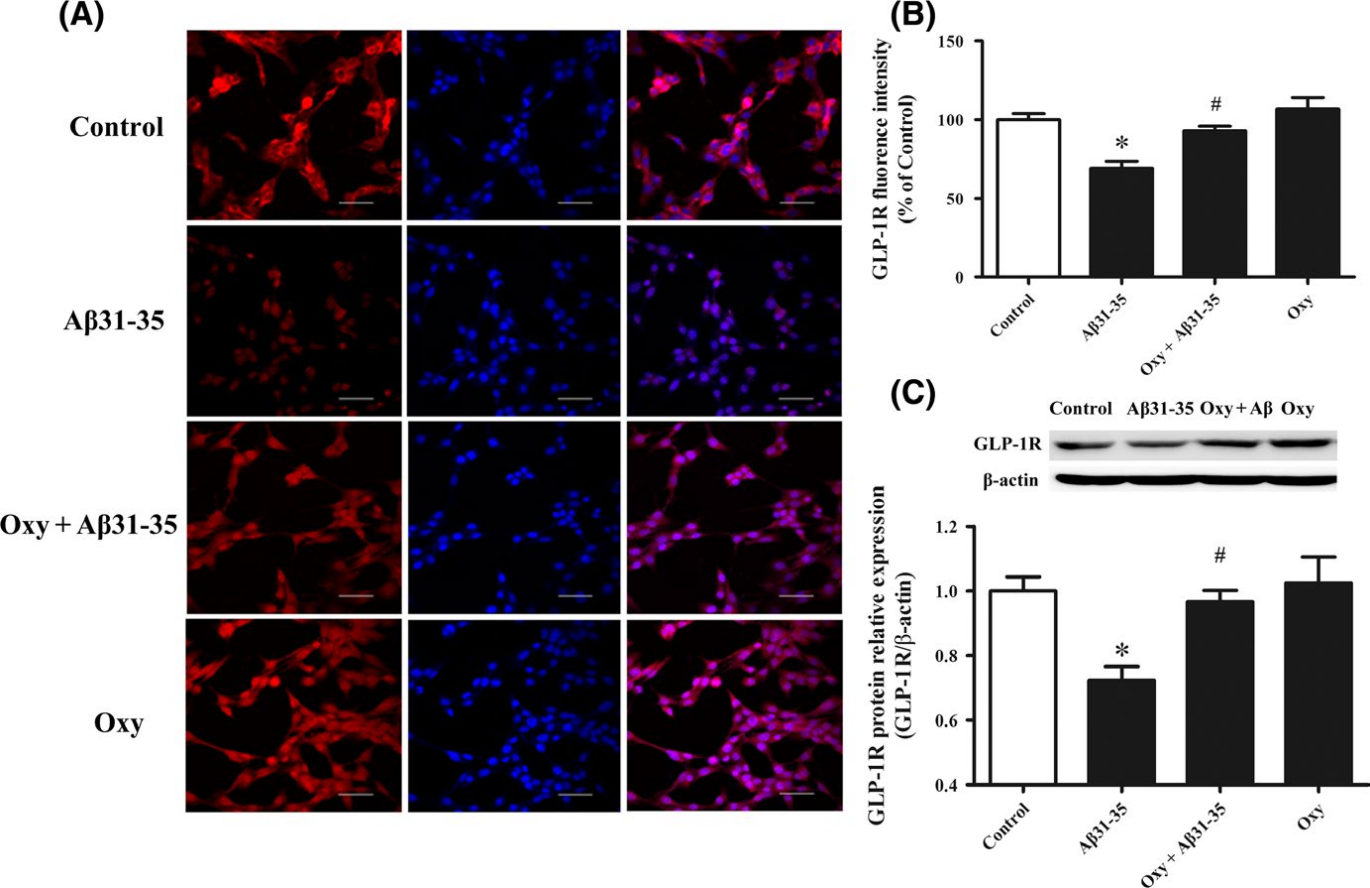
The role of Oxy on Aβ31‐35‐induced decrease in GLP‐1R expression. A, Representative images of immunofluorescence staining for GLP‐1R (red) and DAPI (blue) in HT22 cells. Scale bar: 50 μm. B, Statistical analysis of the fluorescence intensity of GLP‐1R. C, Western blot analysis of GLP‐1R in HT22 cells. Data were expressed as the mean ± SEM (n = 6 per group). *P < .05 compared to the control group; ^#^ *P* < .05 compared to the Aβ31‐35 group

Compared to the control group, Aβ31‐35 significantly reduced GLP‐1R expression in HT22 cells with immunofluorescence staining, Oxy pretreatment significantly increased GLP‐1R expression (Figure 4A,B). In addition, Western blotting was performed to validate the immunofluorescence results of Oxy on GLP‐1R (Figure 4C). The above results indicated that Oxy reversed the Aβ31‐35‐induced decrease in GLP‐1R expression in hippocampal HT22 cells.

### 3.5 GLP‐1R interacts with Oxy to improve Aβ31‐35‐induced circadian rhythm disorder in mice

**Figure 5:**
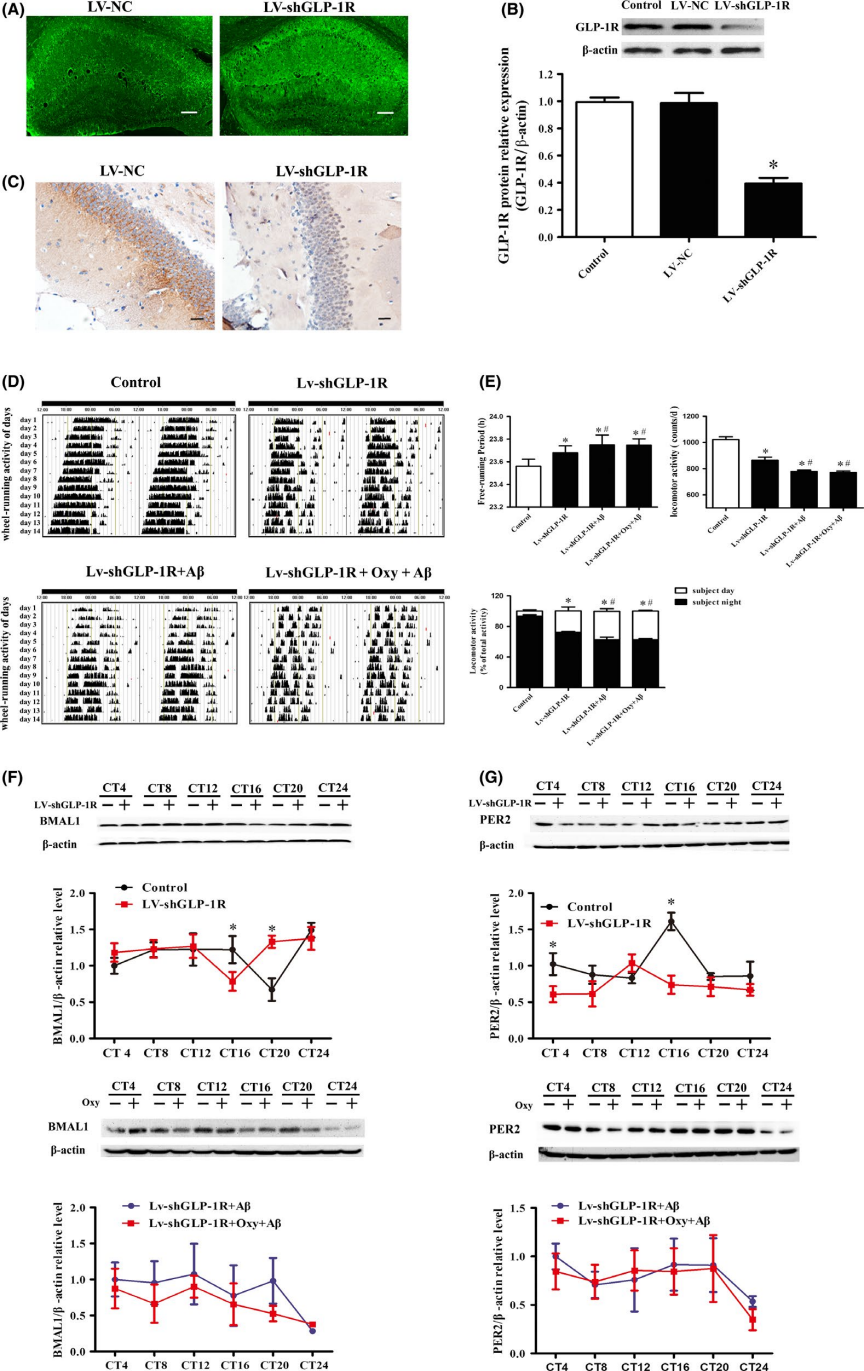
Elucidation of the role of GLP‐1R by interference using lentivirus on Oxy‐improved Aβ31‐35‐induced circadian rhythm disorder in mice. A, Laser scanning confocal microscopy of GFP‐positive cells. Scale bar: 100 μm. B, Western blot analysis of GLP‐1R. C, Immunohistochemical staining for GLP‐1R in the hippocampal CA1 region. Scale bar: 20 μm. D, Representative locomotor activity records of each group. E, The free‐running period of the locomotor activity rhythm, locomotor activity, and the ratio of the subjective night activity/total activity in each group. F, G, Western blotting analysis showing BMAL1 and PER2 protein expression. Data were expressed as the mean ± SEM (n = 6 per group). *P < .05 compared to the control group. ^#^ *P* < .05 compared to the LV‐ShGLP‐1R group

The mice were randomly divided into the control, negative control virus (LV‐NC), and virus (LV‐shGLP‐1R) groups. Hippocampal tissue sections were observed under a laser scanning confocal microscope. GFP‐positive cells were observed in the LV‐shGLP‐1R and LV‐NC groups (Figure 5A). Western blot was used to detect the efficiency of the virus silencing of the target gene GLP‐1R. Compared to the control and the LV‐NC groups, LV‐shGLP‐1R treatment significantly reduced the GLP‐1R protein expression in the hippocampus (Figure 5B). Immunohistochemical staining for GLP‐1R (brown color) showed similar results (Figure 5C). Subsequently, the mice involved in the wheel‐running test that underwent lentivirus‐mediated GLP‐1R interference demonstrated a fragmentation of their movements and an irregular daily sleep‐wake cycle (Figure 5D). Their free‐running period was prolonged, locomotor activity decreased, and subjective night activity/total activity ratio was reduced compared to the control group (Figure 5E). The combination of Aβ31‐35 and GLP‐1R RNA interference caused a more pronounced circadian rhythm disorder, and Oxy could not improve the Aβ31‐35‐induced circadian rhythm disorder in mice after lentivirus‐mediated GLP‐1R RNA interference (Figure 5D,E). We further examined the expression of BMAL1 and PER2 proteins in the hippocampus of each group of mice. The results showed that compared with the control group, the expression of BMAL1 protein in lentivirus‐mediated GLP‐1R interference mice significantly decreased at CT16 and significantly increased at CT20. The expression of the PER2 protein in the lentivirus‐mediated GLP‐1R interference mice significantly decreased at CT4 and CT16. Compared with the combination of Aβ31‐35 and GLP‐1R RNA interference, Oxy did not improve the Aβ31‐35‐induced changes in BMAL1 and PER2 protein expression in the mice after lentivirus‐mediated GLP‐1R RNA interference (Figure 5F,G). These results show that GLP‐1R is required for the Oxy ameliorated Aβ31‐35‐induced disruption of the circadian rhythm.

### 3.6 GLP‐1R plays a role in Aβ31‐35‐induced abnormal expression of Bmal1 and Per2 in HT22 cells

The HT22 cells that were infected with lentivirus vector LV‐shGLP‐1R were selected with puromycin (5 μg/mL). A blank control and empty virus negative control groups were also established. Laser scanning confocal microscopy revealed that the percentages of GFP‐positive cells in the negative control and LV‐shGLP‐1R groups were 81.33 ± 2.04% and 78.30 ± 2.25%, respectively (Figure S1A,B). In addition, Western blotting indicated that the expression of GLP‐1R in the LV‐shGLP‐1R group significantly decreased and no difference was observed between the negative control and blank control groups (Figure S1C). These results suggest that the lentivirus effectively reduce GLP‐1R expression.

**Figure 6:**
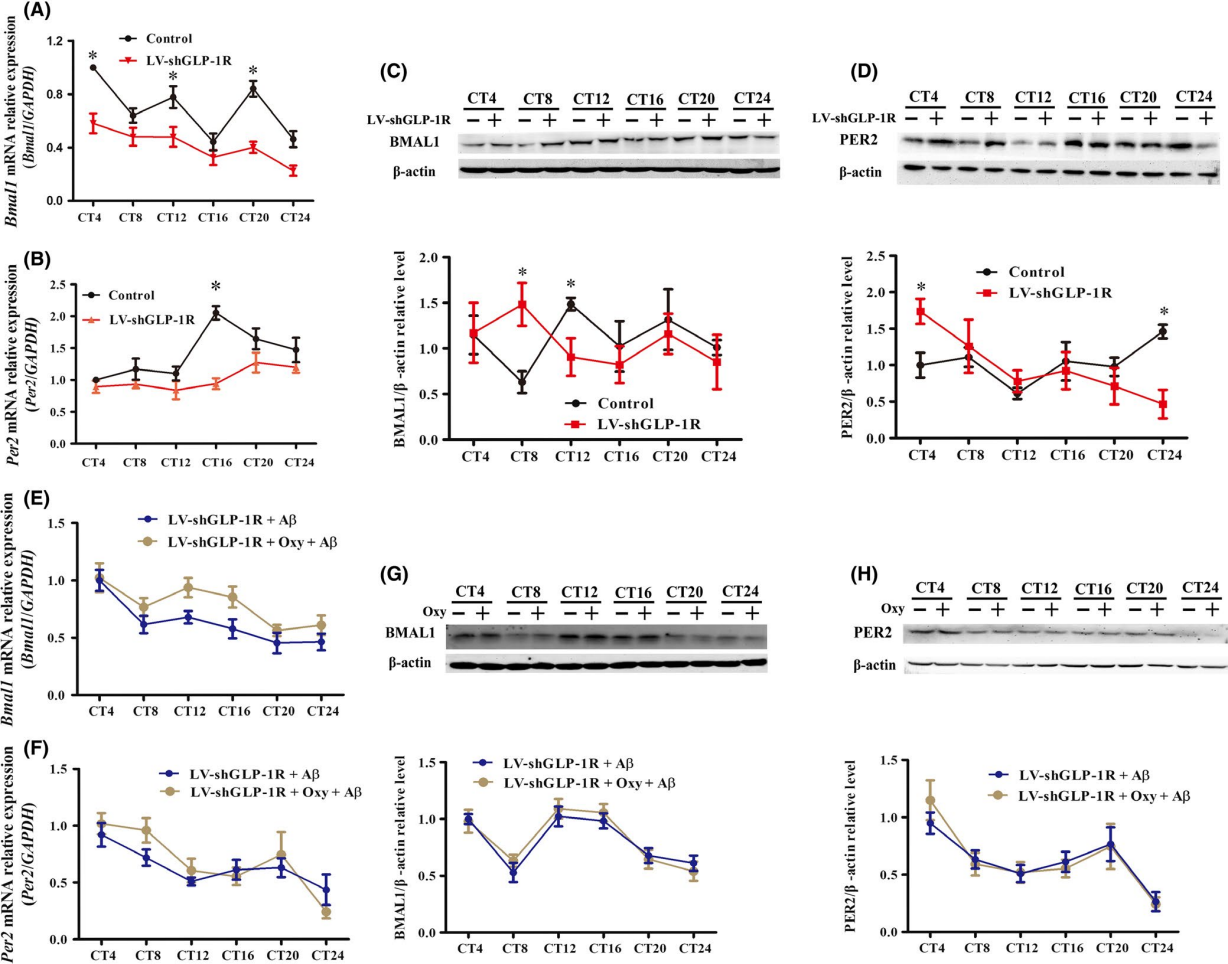
The effect of GLP‐1R interference on Oxy‐improved abnormal expression of Bmal1 and per2 in hippocampal HT22 cells using a lentivirus. A, B, Real‐time PCR was used to measure *Bmal1* and *per2* mRNA expression at indicated circadian times in the control and LV‐shGLP‐1R groups (n = 6 per group). C, D, Western blot analysis of BMAL1 expression and PER2 expression in HT22 cells (n = 8 per group). E, F, mRNA levels of *Bmal1* and *Per2* were assessed at the indicated circadian times in the LV‐shGLP‐1R + Aβ31‐35 and the LV‐shGLP‐1R + Oxy + Aβ31‐35 groups (n = 9 per group). G, H, Western blot analysis of BMAL1 and PER2 at the indicated circadian times in the two groups (n = 9 per group). Data were expressed as the mean ± SEM. *P < .05 compared to the control group

Compared to the negative control group, the *Bmal1* mRNA expression in the LV‐shGLP‐1R group was downregulated at CT4, CT12, and CT20 (Figure 6A). BMAL1 protein expression in the LV‐shGLP‐1R group increased at CT8 and decreased at CT12 compared with the control group (Figure 6C). *Per2* mRNA expression in the LV‐shGLP‐1R group was significantly lower than the negative control group at CT16 (Figure 6B), and PER2 protein expression in the LV‐shGLP‐1R group was increased at CT4 and decreased at CT24 compared with the control group (Figure 6D). Furthermore, Aβ31‐35 and/or Oxy were used to interfere with LV‐shGLP‐1R lentivirus‐infected cells and no significant difference in the Bmal1 and Per2 expression levels was observed between the Aβ31‐35 and Oxy pretreatment groups, indicating that Oxy does not improve the abnormal circadian rhythm of HT22 cells after downregulating GLP‐1R (Figure 6E‐H). These results suggest that GLP‐1R is involved in the pathway in which Oxy improves the expression of clock genes in HT22 cells.

## 4 DISCUSSION

This study has confirmed that Oxy, a new GLP‐1R/GCGR dual receptor agonist, improves circadian rhythm disorders and the Aβ31‐35‐induced abnormal expression of the clock genes Bmal1 and Per2 in the hippocampus and HT22 cells. Using a lentivirus encoding an shGLP‐1R‐GFP‐PURO to interfere with the GLP‐1R gene, we found that GLP‐1R plays an important role in Oxy‐modified circadian rhythm disorders and the Aβ31‐35‐induced abnormal expression of clock genes.

The abnormal deposition of Aβ, excessive phosphorylation of tau protein, and the loss of nerve cells are typical pathological changes involving AD.^16^ Aβ could trigger a pathological cascade in the brain and aggravate other changes that are considered the central link in the formation and development of AD.^17^ Evidence has suggested that the occurrence of AD circadian rhythm disorder is strongly related to the deposition of Aβ in the brain.^3^ The double transgenic AD mice with extensive Aβ deposits in the brain show sleep‐wake cycle disorders.^18^ The results of the present study also suggest that a disruption of the circadian rhythm after intrahippocampal injection of Aβ31‐35, which was expressed as irregular sleep‐wake circadian cycles, sleep fragmentation, prolonged free‐running period, decreased locomotor activity, and a decreased subjective night activity/total activity ratio.

SCN, as a circadian rhythm center of mammals, could integrate the external stimulation of light and food with endogenous rhythms, resulting in an output diurnal rhythm with a period of approximately 24 hours continuously that regulate the circadian changes of a series of physiological activities.^19^ The output of this rhythm is currently believed to be mainly dependent on a transcription‐translation feedback loop (TTFL) consisting of a series of clock genes and clock‐controlled genes. As a core component of TTFL, Bmal1 and Per2 have been shown to be closely related to the maintenance of circadian rhythms. Studies have shown that once Bmal1‐deficient mice migrated from a 12:12 light‐dark environment to a persistently dark environment, they exhibit disordered endogenous motor rhythms due to the loss of light entrainment factors.^20^ Per2^−/−^ mice also showed a marked disruption of running activity rhythm.^21^ Thus, Bmal1 and Per2 play a crucial role in the maintenance of biological circadian rhythms. Although the pacemaker of the circadian oscillation system is located at the SCN, other parts of the brain and peripheral tissues have their own circadian rhythm.^22^ The hippocampus is the main area of Aβ deposition, and it is also one of the important parts reflecting changes in circadian rhythm. The rhythmic oscillation of the circadian clock gene in the hippocampus is called the subordinate oscillator of SCN,^23^ and Aβ induces the degradation of BMAL1 protein, which leads to the destruction of Per2 protein and mRNA oscillation.^4^ Rhythmic expression of clock genes such as Bmal1, Clock, Per, and Cry in the hippocampus of 3 × Tg‐AD mice is significantly impaired.^24^ Some studies have detected gene rhythm changes once every 6 hours.^25^ In previous studies, we also used 6 hours to reflect the basic rhythm changes of clock genes.^26^ There are also studies that have detected gene rhythm changes once every 4 hours,^27^, ^28^ and the addition of time points can more accurately reflect rhythmic changes. Therefore, we selected the 4‐hour interval to reflect rhythmic in the expression of clock genes. In this study, we found that the core clock genes Bmal1 and Per2 in the hippocampus of mice exhibit distinct circadian oscillations. A previous study has described a significant reduction of the Bmal1 and Per2 expression in the SCN of 5XFAD mice with excessive Aβ deposition in the brain.^4^ Our results showed that Aβ31‐35 caused the abnormal expression of Bmal1 and Per2 in mouse hippocampus, which agrees with previous reports.^4^ In addition, we found that the Bmal1 and Per2 in HT22 cells also exhibited rhythmic oscillations. However, after the intervention of Aβ31‐35, HT22 cells showed arrhythmic Bmal1 and Per2 expression. Taken together, these findings indicate that Aβ31‐35 could disrupt the circadian rhythm, as indicated by the irregular wheel‐running activity and arrhythmic oscillations of the clock genes in the mouse hippocampus and HT22 cells.

The correlation between AD and type 2 diabetes mellitus (T2DM) was first reported in 2004.^29^ An epidemiological survey showed that 85% of AD patients have impaired fasting glycemia (IFG) or even T2DM. Meanwhile, the chronic complications of T2DM could cause brain lesions and increase AD risk.^6^ Interestingly, AD and T2DM have pathological similarities such as amyloid plaque deposition and tau protein hyperphosphorylation.^30^ In addition, streptozotocin (STZ) could induce diabetes in AD models, which in turn provide important etiological evidence for the correlation of the two diseases.^31^ GLP‐1 derivatives as T2DM therapeutic drugs could improve the memory and cognition of AD patients.^32^ OXM could simultaneously activate both GLP‐1R and GCGR and is a more efficacious therapeutic regimen for diabetes than GLP‐1R agonists.^8^ A recent study showed that Oxy which is an analogue of OXM provides a significant improvement in the dyskinesia of MPTP‐treated PD mice.^11^ In addition, the administration of Oxy to high‐fat fed rats resulted in a significant reduction in oxidative stress in the hippocampus and promoted synapse formation in both the cortex and hippocampus via the insulin signaling pathway.^33^ In the present study, H&E staining was used to assess morphological changes in the hippocampus CA1 neurons. We observed that the neurons were loosely arranged, and the number of neurons decreased after Aβ31‐35 treatment. Pretreatment with Oxy alleviated Aβ31‐35‐induced neuronal damage (Figure S2). We further tested the toxicity of Oxy using the CCK‐8 assay, and the results showed that Oxy (100 nmol/L) significantly reversed the decreased viability of HT22 cells that was induced by Aβ31‐35 (5 μmol/L), suggesting that Oxy has a cytoprotective effect (Figure S3). Then, the present study determined that Oxy‐pretreated mice exhibited a recovered sleep‐activity pattern and improved circadian rhythm disorder compared to Aβ31‐35‐treated mice. Oxy could antagonize the Aβ31‐35‐induced abnormal expression of Bmal1 and Per2 in the hippocampus or HT22 cells. Intrahippocampal injection is an invasive procedure, so we chose to inject the subjects only once; and Oxy was administered in the intrahippocampal space before Aβ. Results showed that Oxy pretreatment could antagonize the toxic effects of Aβ at an early stage, and we are pleased to find that the early antagonism of Oxy could achieve a continuous improvement in circadian rhythm disorder induced by Aβ. Similarly, some studies have found that intrahippocampal injection of the GLP‐1 analogue Liraglutide prevented Aβ‐induced impairment of spatial learning and memory.^34^ Therefore, we believe that the one‐time intrahippocampal injection of Oxy could effectively improve Aβ‐induced circadian rhythm disorders and abnormal clock gene expression.

The expression of GLP‐1R in the mouse brain is higher than that of GCGR.^13^ Therefore, we hypothesized that the effect of Oxy improves Aβ31‐35‐induced circadian rhythm disorder in mice, and the abnormal expression of clock genes in the HT22 cells is mainly dependent on the activation of GLP‐1R, a specific binding receptor for GLP‐1 that is also widely distributed in the central nervous system. During showed that GLP‐1R^−/−^ mice exhibit a severely impaired learning ability and significantly decreased synaptic plasticity. Furthermore, the learning ability deficiency could be restored by hippocampal GLP‐1R gene transfection.^35^ This study confirmed that Aβ31‐35 could downregulate the expression of GLP‐1R in HT22 cells, whereas Oxy could antagonize the Aβ31‐35‐induced reduction in GLP‐1R expression in HT22 cells. To further verify the role of GLP‐1R in improving Aβ31‐35‐induced circadian rhythm disorders with Oxy, we used a lentiviral vector to interfere with the GLP‐1R gene. The results showed that the inhibition of the GLP‐1R expression in the mouse hippocampus could lead to a disruption of the circadian rhythm and the expression of BMAL1 and PER2; the treated mice showed irregular daily sleep‐wake cycles, a prolonged free‐running period, reduced locomotor activity, and decreased ratio of subjective night activity to total activity. Aβ31‐35 further aggravated the GLP‐1R RNA interference‐induced circadian rhythm disorder and the abnormal expression of BMAL1 and PER2. After further silencing GLP‐1R in the HT22 cells, we found that the expression levels of Bmal1 and Per2 did not exhibit regular circadian oscillations in the HT22 cells, indicating that the GLP‐1R activation enhances the rhythmic expression of clock genes to a certain extent. Furthermore, we also found that the effect of the Oxy‐antagonized Aβ31‐35‐induced change in clock gene expression was significantly weaker in the HT22 cells after GLP‐1R interference. These results suggest that GLP‐1R plays an important role in the regulation of the circadian rhythm of Oxy.

## 5 CONCLUSION

In summary, this study demonstrated for the first time that Oxy could improve Aβ31‐35‐induced circadian rhythm disorders and the abnormal expression of Bmal1 and Per2 clock genes in mice and HT22 cells. It further clarified that GLP‐1R plays a critical role in the maintenance of the circadian rhythm and rhythmic oscillation of Bmal1 and Per2. This study provides a novel target for the prevention and treatment of patients with circadian rhythm disorders.

## CONFLICTS OF INTEREST

The author reports no potential conflict of interests.
