# Supplementary figures and images for: D‐Ser2‐oxyntomodulin ameliorated Aβ31‐35‐induced circadian rhythm disorder in mice
Source: CNS Neurosci Ther. 2019 Aug 14;26(3):343–54. doi: 10.1111/cns.13211 (PMC7053239; doi:10.1111/cns.13211)

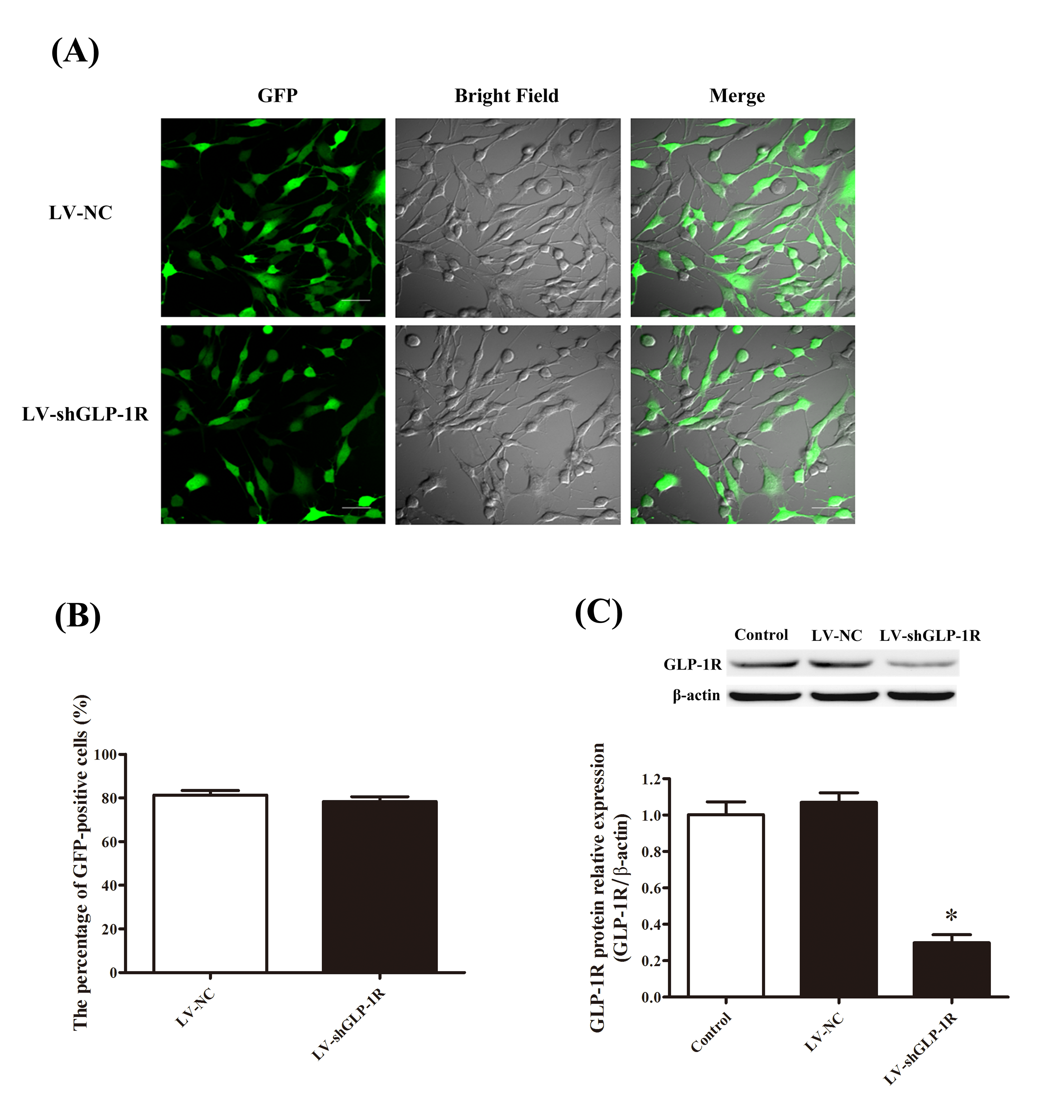

Supplement: Supplementary file 1 [file CNS-26-343-s001.tif]

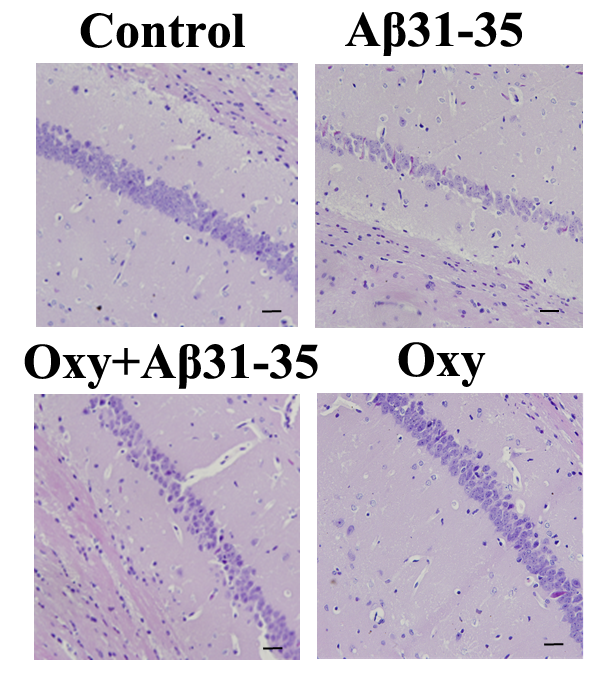

Supplement: Supplementary file 2 [file CNS-26-343-s002.tif]

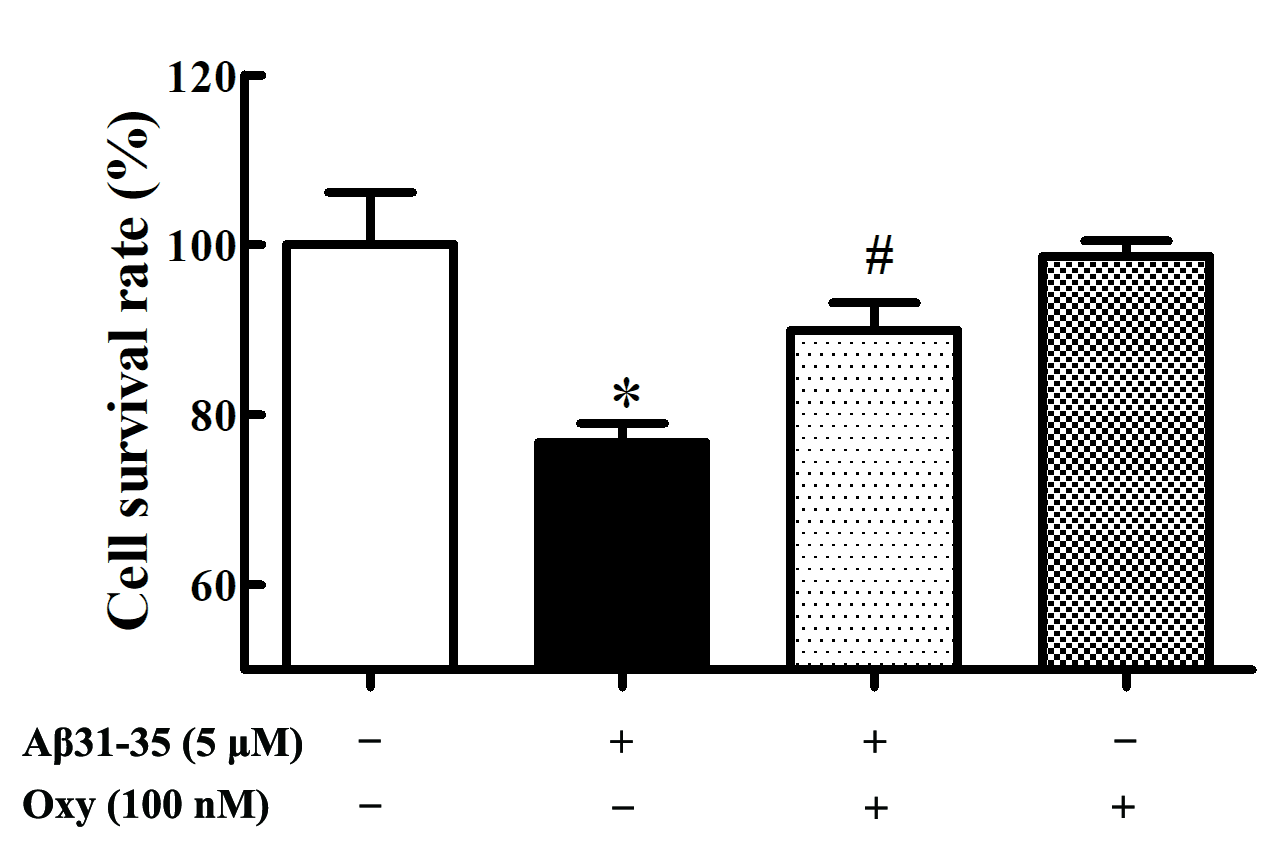

Supplement: Supplementary file 3 [file CNS-26-343-s003.tif]
