# Supplementary material for: D‐Ser2‐oxyntomodulin ameliorated Aβ31‐35‐induced circadian rhythm disorder in mice
Source: CNS Neurosci Ther. 2019 Aug 14;26(3):343–54. doi: 10.1111/cns.13211 (PMC7053239; doi:10.1111/cns.13211)
Supplement: Supplementary file 4 [file CNS-26-343-s004.doc]

**Supplementary methods**

1. **H&E Staining**

After the end of the mouse wheel-running behavioral experiment, the brain tissue was dissected and fixed with 10% neutral formalin solution, followed by paraffin embedding and tissue sectioning. The tissue sections were then dried in an oven, deparaffinized in a xylene solution, and dehydrated through an ethanol gradient. The dried tissue sections were stained with 0.1% hematoxylin for 5 min, then rinsed in running water for 10 min and then with distilled water for a few seconds. After rinsing with 95% ethanol for 5 s, the sections were stained with 0.5% eosin for 30 s and dried across an ethanol gradient, mounted in neutral gum, and examined under a light microscope.

1. **CCK-8 assay**

In this study, cell viability was detected by the CCK8 assay as follows: The cell suspension (100 μL/well) was seeded into 96-well microplates, and incubated overnight to allow the cells to adhere to the bottom of the wells. Next, the cells were treated accordingly. After 24 h, 10 µL of the CCK-8 reagent were added to each well, and the plates were incubated for 1.5-2 h. Finally, the optical density (OD) of each group was measured at a wavelength of 450 nm with a microplate reader. The cell survival rate of each treatment group compared to the normal control group was calculated based on the OD values of each group using the following equation: Cell survival rate = OD value (Treatment group) - OD value (Blank control group) / OD value (Control group) - OD value (Blank control group) × 100%.

**Supplementary Figure Legends**

**Supplementary Figure S1 The expression of GLP-1R after GLP-1R interference of HT22 cells using a lentivirus.** (A) Confocal laser-scanning microscopy detection of GLP-1R (green) in HT22 cells. Scale bar: 40 μm. (B) Quantification of the percentage of GFP-positive cells. (C) Western blot analysis of GLP-1R in each group. Control: blank control group; LV-NC: empty virus negative-control group; LV-shGLP-1R: lentivirus encoding an shGLP-1R-GFP-PURO group. Data were expressed as the mean ± SEM (n = 6 per group). * P<0.05 compared to the blank control group.

**Supplementary Figure S2 Effect of Aβ31-35 and Oxy treatment on the CA1 region of the hippocampus (H&E staining, Scale bar: 20 μm).**

**Supplementary Figure S3 The effect of 5 μM Aβ31-35 and pre-treatment with 100 nM Oxy on HT22 cells after 24 h using the CCK-8 assay.** Data are presented as means ± SEM (n = 10). * P<0.05 compared to the control group; ^#^ P<0.05 compared to the Aβ31-35 group.
